# Supplementary material for: Identification of photoperiod-induced specific miRNAs in the adrenal glands of Sunite sheep (Ovis aries)
Source: Front Vet Sci. 2022 Jul 22;9:888207. doi: 10.3389/fvets.2022.888207 (PMC9354845; doi:10.3389/fvets.2022.888207)
Supplement: Supplementary file 1 [file Data_Sheet_1.ZIP › Supplementary file/miRDeep2 file.zip!/miRDeep2 file/Know_Structure/LP42A3/oar-mir-1197.pdf]

miRBase precursor : oar-mir-1197
Total read count : 136
oar-miR-1197-5p read count : 0
oar-miR-1197-3p read count : 113
remaining reads : 23

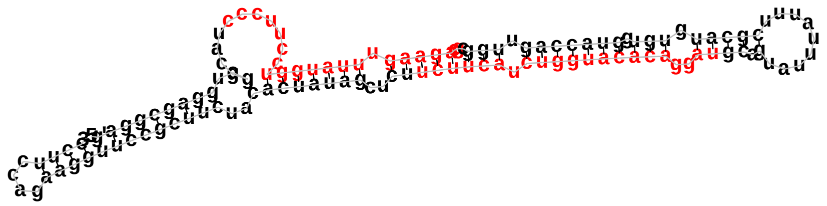

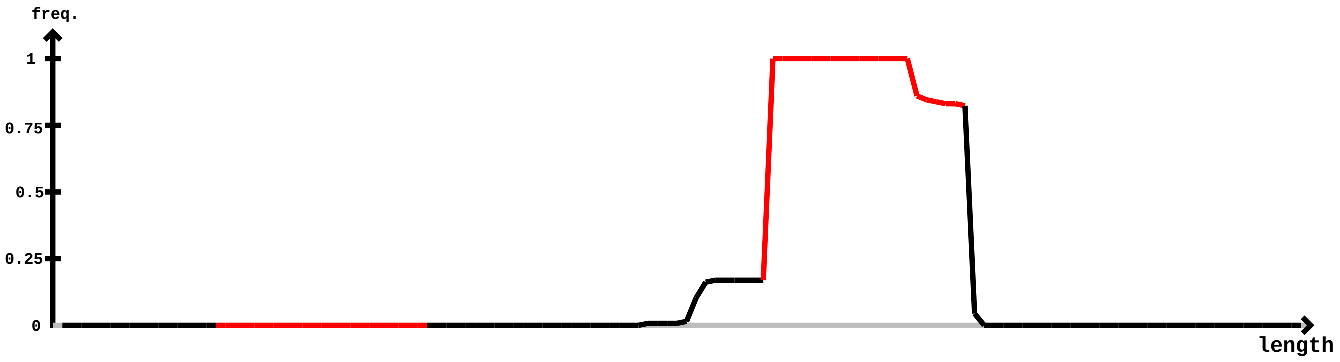


```
                                                                         oar-miR-1197-3p
                oar-miR-1197-5p
5'- gaggcgaggucggcaucccuuccugguauuugaagacgcgguugaccaugguguguacgcuuuauuuaugacguaggacacauggucuacuucuucucgauaucacaucuucgccuuggaagaccuucc   -3'    exp
    ((((((((..(............(((((((.(((((...(((.(((((((.(((.(((((.........).))))..)))))))))).))))))))..))))))))..)))))))))((((....))))    reads     mm
    ............................................................uuuauuuaugacguaggacacauggucu.........................................    1         0
    ................................................................uuuaugacguaggacacauggucu.........................................    1         0
    .................................................................uuaugacguaggacacauggucu.........................................    12        0
    ..................................................................uaugacguaggacacauggucu.........................................    5         0
    ..................................................................uaugacguaggacacauggucua........................................    1         0
    ..................................................................uaugacguaggacacauggucuac.......................................    1         0
    ..................................................................uaugacguaggacacauggucuacu......................................    1         0
    ...................................................................augacguaggacacauggucua........................................    1         0
    .........................................................................uaggacacauggucuacuuc....................................    1         0
    .........................................................................uaggacacauggucuacuucu...................................    106       0
    .........................................................................uaggacacauggucuacuucuu..................................    6         0
```
